# Transient Extensive Portal Venous Gas in a Dialysis Patient

**DOI:** 10.7759/cureus.95535

**Published:** 2025-10-27

**Authors:** Michael J Martinez, John J Murphy, Francisco R Halili

**Affiliations:** 1 Radiology, St. Matthew's University, Grand Cayman, CYM; 2 Radiology, Orlando Health St. Cloud Hospital, St. Cloud, USA; 3 General Surgery, Orlando Health St. Cloud Hospital, St. Cloud, USA

**Keywords:** abdominal pain, computed tomography, diagnostic laparoscopy, end-stage renal disease, hemodialysis, hepatic portal venous gas, mesenteric ischemia, portal venous gas

## Abstract

Hepatic portal venous gas (HPVG) is a serious radiologic indicator that can suggest bowel ischemia and lead to fatal outcomes. The medical field now identifies transient HPVG without ischemia more often because CT technology has spread throughout the healthcare system. We report a 70-year-old woman with end-stage renal disease (ESRD) on hemodialysis who developed acute abdominal pain during dialysis. The CT scan results showed HPVG extending into the superior mesenteric venous system. The medical team performed an urgent diagnostic laparoscopy because of ischemia concerns, which showed that the bowel tissue remained healthy without signs of necrosis or perforation. She improved clinically with supportive care, and at 48 hours, a repeat CT showed complete resolution of HPVG. The case illustrates that medical imaging results need to match clinical observations and surgical findings to determine which patients can receive nonsurgical treatment.

## Introduction

Hepatic portal venous gas (HPVG) is defined as the presence of gas within the portal venous system. Historically, HPVG was considered a radiologic harbinger of catastrophic intra-abdominal pathology - most notably mesenteric ischemia - associated with high mortality [1,2]. With widespread multidetector CT, HPVG is now recognized as a context-dependent finding that can arise from benign or self-limited conditions when interpreted alongside clinical status and imaging markers [3-5]. Contemporary management, therefore, emphasizes integrating clinical status, laboratory markers (e.g., lactate), and CT indicators of ischemia rather than viewing HPVG itself as an automatic mandate for surgery [3-5].

Consistent with prior literature, several risk contexts are described, including ischemic/low-flow states, mucosal injury from inflammation or infection that facilitates gas translocation, iatrogenic/post-procedural sources, and increased intraluminal pressure driving luminal gas across compromised mucosa into mesenteric veins [3-5]. In the modern CT era, a subset of HPVG presentations is benign when clinical and imaging findings are reassuring, whereas poor prognosis tends to cluster with peritonitis, hemodynamic instability, lactic acidosis, and ischemic CT features such as pneumatosis, absent mural enhancement, or portomesenteric venous thrombosis [3-5]. Among patients receiving hemodialysis, reports include both benign, transient HPVG and severe presentations associated with peritonitis, pneumatosis, or hypotension [6-9]. We present a case of extensive, transient HPVG detected during hemodialysis that resolved with conservative therapy after a negative diagnostic laparoscopy. This paradigm shift underscores the role of multidisciplinary evaluation - particularly in at-risk populations such as patients undergoing hemodialysis - so that radiologic findings are weighed with clinical/laboratory data and CT indicators of ischemia [3-5,8].

## Case presentation

A 70-year-old woman with end-stage renal disease (ESRD) on thrice-weekly hemodialysis developed acute, diffuse abdominal pain with associated nausea during a dialysis session. She denied fever, hematemesis, hematochezia, and diarrhea. There was no history suggestive of recent endoscopy or abdominal trauma.

Examination

On presentation, she was afebrile, with a blood pressure of 128/56 mmHg, heart rate of 68 bpm, and oxygen saturation of 98% on room air. The abdomen was distended and diffusely tender without guarding or rebound. No peritoneal signs were present.

Laboratory findings

Admission laboratory results are summarized in Table 1. Notably, her white blood cell count (WBC) was within reference range, lactate was within normal limits, and there was no biochemical evidence of metabolic acidosis - features that do not support mesenteric ischemia in the appropriate clinical and imaging context - consistent with prior reports [3-5,8].

**Table 1:** Laboratory findings on presentation. WBC: white blood cell count; BUN: blood urea nitrogen

Parameter	Result	Units	Reference Range
WBC	9.2	10^9^/L	4.0-10.0
Hemoglobin	9.9	g/dL	12.0-16.0
Platelet count	151	10^9^/L	150-450
Lactate	2	mmol/L	0.5-2.2
BUN	47	mg/dL	7-20
Serum creatinine	6.2	mg/dL	0.6-1.2

Imaging

**Figure 1:**
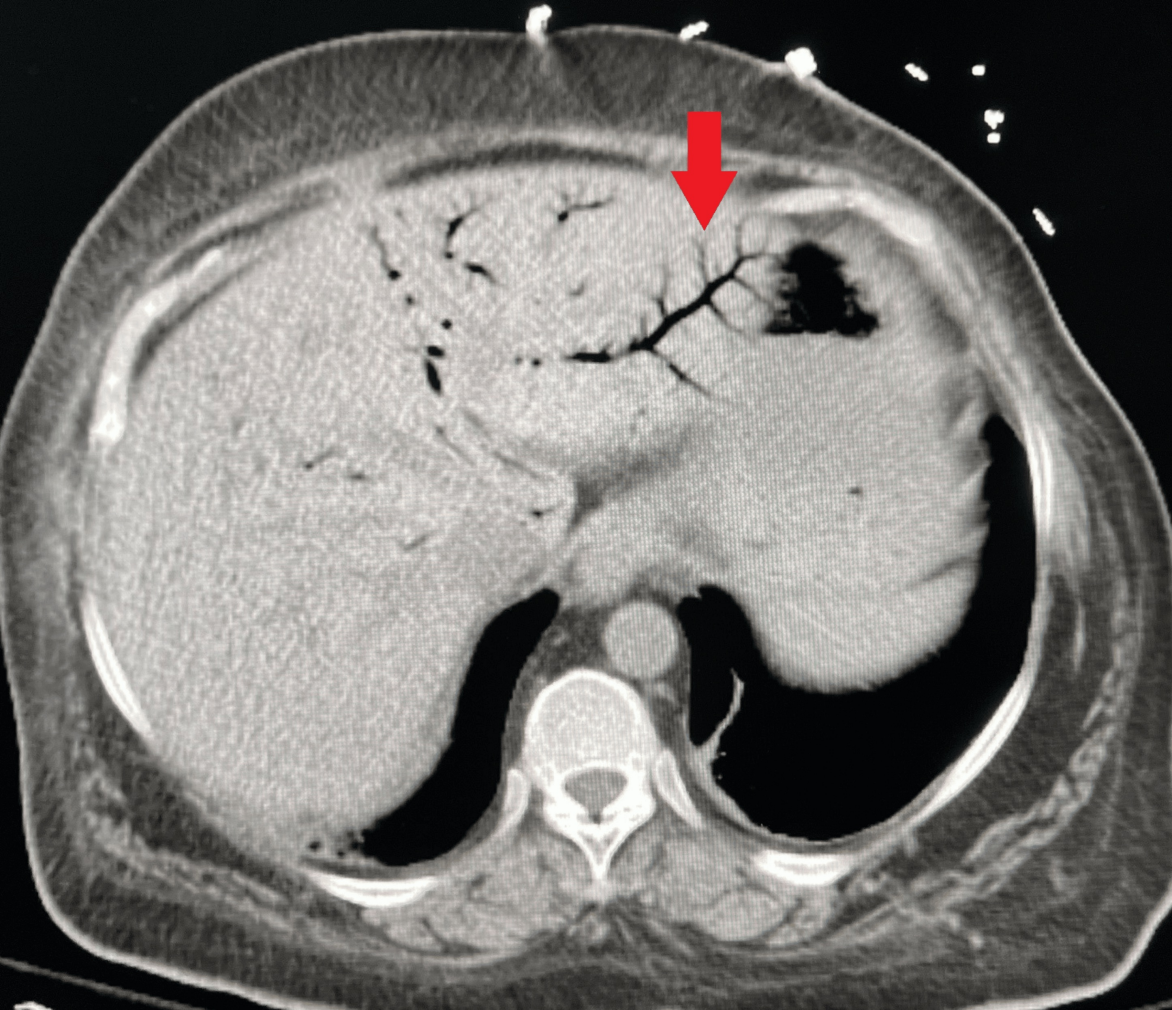
Axial CT of the abdomen demonstrating extensive HPVG, seen as branching hypodensities extending to the periphery of the liver (red arrow). HPVG: hepatic portal venous gas

CT of the abdomen and pelvis demonstrated extensive HPVG throughout the liver with branching peripheral hypodensities, as well as gas within the superior mesenteric vein. There was no evidence of pneumoperitoneum, bowel wall discontinuity, or portomesenteric venous thrombosis (Figure 1). CT imaging was obtained promptly after presentation, and due to concern for mesenteric ischemia, the patient underwent urgent diagnostic laparoscopy the same day. Intraoperative inspection revealed globally viable small and large bowel without necrosis, ischemia, perforation, or discoloration. A Blake drain (Ethicon, Somerville, USA) was placed.

Hospital course

**Figure 2:**
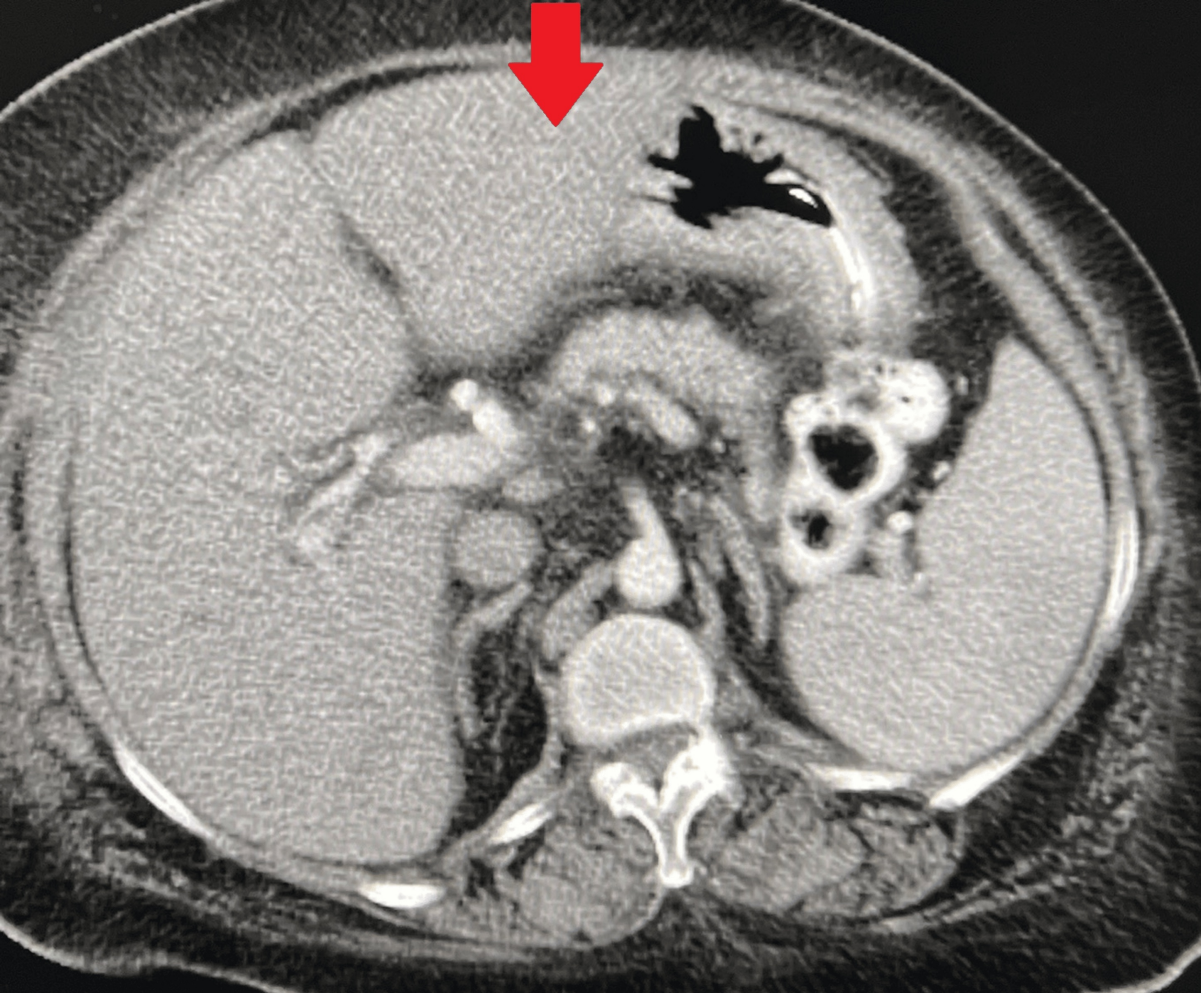
Follow-up axial CT obtained 48 hours later demonstrating complete resolution of HPVG. The red arrow indicates the same peripheral hepatic region previously involved, now without portal venous gas. HPVG: hepatic portal venous gas

Postoperatively, the patient received intravenous fluids, broad-spectrum antibiotics, bowel rest, and scheduled hemodialysis. Vital signs remained stable without clinical deterioration throughout hospitalization. A follow-up CT at 48 hours demonstrated complete resolution of HPVG (Figure 2). The patient became pain-free with diet advancement and was discharged in stable condition.

## Discussion

HPVG has traditionally been regarded as a marker of catastrophic intra-abdominal disease - most notably mesenteric ischemia - with early series emphasizing frequent association with bowel necrosis and high mortality [1,2]. With widespread CT use, however, a broader and partly benign spectrum of causes has been recognized, and HPVG alone no longer mandates emergent laparotomy in every case [3,4]. Reassuring clinical status, normal or near-normal lactate, and absence of ischemic CT markers identify patients who can often be treated nonoperatively with close monitoring and supportive care [3-5,8]. In our patient, there was no leukocytosis, lactate was within normal limits, and CT lacked ischemic features - findings inconsistent with mesenteric ischemia in this context [3-5,8].

The pathophysiology of HPVG is commonly attributed to mucosal disruption and/or elevated intraluminal pressure that permits luminal or bacterial gas to enter mesenteric veins and the portal system [5]. In hemodialysis, rapid fluid shifts and transient perfusion variability may compromise mucosal integrity and facilitate gas migration, a plausible mechanism consistent with prior reports of dialysis-associated HPVG [6,9]. Within the hemodialysis population, published cases illustrate both a benign, transient phenotype that resolves with conservative therapy when clinical and CT indicators of ischemia are absent and a more severe phenotype associated with peritonitis, hypotension, elevated lactate, or pneumatosis [6-9].

Our patient had extensive HPVG and gas within the superior mesenteric vein but no peritoneal signs, no pneumatosis or portomesenteric thrombosis, and lactate within normal limits. Diagnostic laparoscopy confirmed globally viable bowel, and HPVG resolved completely within 48 hours under conservative management (bowel rest, antibiotics, hemodynamic optimization, and scheduled dialysis). These findings align with proposed approaches supporting nonoperative care in stable, low-risk presentations [3,4,8]. In patients with ESRD, individualized risk stratification that considers transient perfusion changes during dialysis can help distinguish benign, reversible HPVG from true ischemic pathology [3-5,8].

Authors' perspective

Practically, a multidisciplinary pathway (radiology, surgery, nephrology) that (i) assesses hemodynamics and peritoneal signs, (ii) scrutinizes CT for ischemic markers, (iii) integrates lactate and other labs for risk stratification, and (iv) reserves surgery for instability, peritonitis, ischemic CT features, or strong biochemical evidence of ischemia, is consistent with existing literature and may reduce nontherapeutic laparotomy while maintaining timely intervention for true ischemia [3-5,8].

## Conclusions

Extensive HPVG on CT can be alarming but is not invariably catastrophic, particularly in hemodynamically stable patients without ischemic indicators. In hemodialysis patients, transient HPVG may arise from low-flow states and mucosal injury and can respond to conservative care. Management should be guided by an integrated assessment of clinical status, laboratory markers (e.g., lactate), and CT features, with surgery reserved for patients who are unstable, have peritonitis, or demonstrate imaging or intraoperative evidence of bowel ischemia. Optimal management integrates clinical, laboratory, and imaging findings to guide intervention, avoiding unnecessary laparotomy while ensuring timely surgery in cases of true ischemia, including scenarios in which transient perfusion changes during dialysis may mimic ischemic pathology.
